# Factors associated with a malaria outbreak at Tongogara refugee camp in Chipinge District, Zimbabwe, 2021: a case–control study

**DOI:** 10.1186/s12936-022-04106-9

**Published:** 2022-03-19

**Authors:** Kudzai Patience Takarinda, Simon Nyadundu, Emmanuel Govha, Notion Tafara Gombe, Addmore Chadambuka, Tsitsi Juru, Mufuta Tshimanga

**Affiliations:** 1 Department of Primary Health Care Sciences, Global and Public Health Unit, University of Zimbabwe, Harare, Zimbabwe; 2 Provincial Medical Directorate, Zimbabwe Ministry of Health and Child Care, Manicaland , Zimbabwe; 3 African Field Epidemiology Network, Harare, Zimbabwe; 4 Zimbabwe Field Epidemiology Training Health Studies Office, Office 3-68 Kaguvi Building, Harare, Zimbabwe

**Keywords:** Malaria outbreak, Tongogara refugee camp, Refugee housing unit (RHU), Zimbabwe

## Abstract

**Background:**

Malaria is a leading cause of morbidity and mortality among forcibly displaced populations, including refugees, approximately two-thirds of whom reside in malaria endemic regions. Data from the rapid disease notification system (RDNS) reports for Manicaland Province in Zimbabwe showed that despite implementation of malaria control initiatives, there was an increase in number of malaria cases above action thresholds at Tongogara refugee camp in Chipinge district during weeks 12–14 of 2021. An investigation that described the outbreak by person, place and time was conducted. Malaria emergency preparedness, response, and appropriateness of case management were assessed. The factors associated with contracting malaria were determined to enable the formulation of appropriate interventions, establish control, and prevent future malaria outbreaks among this vulnerable population.

**Methods:**

A 1:1 unmatched case–control study involving 80 cases and 80 controls was conducted using interviewer-administered questionnaires at household level. Data was entered into Epi Data version 3.1 and quantitative analysis was done using Epi Info™ version 7.2.2.6 to generate medians, proportions, odds ratios and their 95% confidence intervals.

**Results:**

Malaria cases were distributed throughout the 10 residential sections within Tongogara refugee camp, the majority being from section 7, 28 (35%). Despite constituting 11% of the total population, Mozambican nationals accounted for 36 (45%) cases. Males constituted 47 (59%) among cases which was comparable to controls 43 (54%), p = 0.524. The median age for cases was 15 years [Interquartile range (IQR), 9–26] comparable to controls, which was 17 years (IQR, 10–30) (p = 0.755). Several natural and man-made potential vector breeding sites were observed around the camp. Risk factors associated with contracting malaria were engaging in outdoor activities at night [AOR = 2.74 (95% CI 1.04–7.22), wearing clothes that do not cover the whole body during outdoor activities [AOR 4.26 (95% CI, 1.43–12.68)], while residing in a refugee housing unit reduced the risk of contracting malaria [AOR = 0.18 (CI, 0.06–0.55)].

**Conclusions:**

The malaria outbreak at Tongogara refugee camp reemphasizes the role of behavioural factors in malaria transmission. Intensified health education to address human behaviours that expose residents to malaria, habitat modification, and larviciding to eliminate mosquito breeding sites were recommended.

## Background

Malaria is a life-threatening disease which is preventable and curable. Globally, an estimated 229 million cases of malaria and 409,000 attributable deaths were reported in 2019 [1]. Data has shown that the World Health Organization (WHO) African Region carried a disproportionately high share of the global malaria burden accounting for 94% of malaria cases and deaths in 2019 [2] In Zimbabwe, approximately 310,000 malaria cases were reported in 2019, translating to an incidence rate of 22 cases per 1000 population and a total of 266 deaths [3].

The United Nations Refugee Agency estimates that for the year 2020, there were 79.5 million forcibly displaced people, including 26 million refugees globally [4]. Additionally, malaria is a leading cause of morbidity and mortality among these vulnerable populations, approximately two-thirds (63%) of whom reside in malaria endemic regions [4, 5]. Data on malaria incidence and mortality from the United Nations High Commissioner for Refugees (UNHCR) Health Information System (2006–2009) analysed by Anderson et al. in 2011 showed that an annual average of 111,571 malaria cases were reported among refugees [6]. Children younger than five years of age accounted for 40,410 (36.2%) cases, while, an estimated 12.3% of all refugee deaths and 16% of deaths among refugee children under five years of age were due to malaria [6].

Malaria is an acute febrile illness and symptoms usually appear 10–15 days after the infective mosquito bite, in a non-immune individual [2, 7]. In malaria endemic areas, people may develop partial immunity, allowing asymptomatic infections to occur [8]. However, some population groups are at considerably higher risk of contracting malaria, and developing severe disease, than others. These include children less than 5 years of age, pregnant women and patients with HIV/AIDS, as well as non-immune migrants, mobile populations and travelers [9].

Amongst the causative agents of malaria, *Plasmodium falciparum* accounts for approximately 99.7% of estimated malaria cases in the WHO African Region, including Zimbabwe [2]. The vectors for malaria parasites are the female *Anopheles* mosquitoes, which bite mainly between sunset and sunrise. Malaria is transmitted in tropical and subtropical areas, where high temperatures, humidity and rainfall are critical for the reproduction and growth cycle of the *Anopheles* mosquitoes and malaria parasites [10]. The WHO recommends two core vector control interventions, mainly the use of long-lasting insecticidal nets (LLINs) and indoor residual spraying (IRS) [2]. The Zimbabwe 2016–2020 National Malaria Strategic Plan, deploys IRS in areas with an annual parasite index (API) of 5 per 1000 population or greater such as Chipinge district [11]. Long-lasting insecticidal nets are deployed in areas with an API of 2–4 per 1000 population and there are provisions for the distribution of nets for outdoor sleeping spaces among populations that are prone to sleeping outside during summer [11].

In Zimbabwe, the IRS Programme is a standard annual practice conducted to ensure control and mitigation of malaria epidemics. The malaria early warning system (MEWS) under the Epidemiology and Disease Control Department within the Ministry of Health and Child Care in Manicaland Province conducted IRS during the period October to November in 2020 among other interventions. Weather forecasts by the Meteorological Service Department predicted above normal rainfall, for the 2020/21 rainy season in Zimbabwe [12]. Despite the forecasted weather patterns, data from the rapid disease notification system (RDNS) weekly reports for Manicaland Province for weeks 1–14 showed a 75% decline of malaria cases from 64 130 in 2020 to 16 237 in 2021. Chipinge district also reported a 33% decline of malaria cases from 7466 to 5005 during the same period. However, (TRC) clinic in Chipinge district reported a 123% increase in malaria cases from 75 cases in 2020 to 167 cases in 2021 between weeks 1–14 of 2021. Interventions implemented at TRC to control the spread of malaria included distribution of LLINs, IRS, and health information dissemination. However, TRC clinic recorded 29, 20 and 23 malaria cases above the action threshold levels of 15, 9 and 7 malaria cases, in week 12, 13 and 14 of 2021, respectively.

Several factors promote vulnerability to malaria illness and death among refugees. Understanding the epidemiology of malaria in these vulnerable populations is important to enable the formulation of appropriate interventions to establish control and prevent future episodes of malaria outbreaks within the camp and other areas affected by humanitarian emergencies among displaced populations.

An investigation of the malaria outbreak among residents at Tongogara refugee camp in Chipinge District, Zimbabwe, 2021 was conducted. The specific objectives were to describe the outbreak by person, place and time, determine the factors associated with contracting Malaria, and assess the appropriateness of case management during the malaria outbreak. Additionally, an assessment of malaria emergency preparedness and response as well as the knowledge of malaria among refugees was conducted.

## Methods

### Study design and setting

An unmatched 1:1 case–control study was conducted at TRC. The camp has been in existence since 1983 and was established by the Government of Zimbabwe because of civil unrest in neighboring Mozambique. The open community camp originally designed to cater for a maximum of 3000 people has an estimated population of 15,049 refugees within 10 housing sections and over 200 Zimbabwean nationals working for various organizations as of 30 April 2021.

Males constitute 7999 (53.2%) of the total population while children aged 5–9 years account for the majority of the population 2375 (15.8%) followed by children under 5 years constituting 2125 (14.1%). However, the population is not static as there are new refugee arrivals and departures almost daily. To date, more than three quarters of the population originates from the Democratic Republic of Congo followed by nationals from Mozambique 1644 (11%), Burundi 918 (6%) and Rwanda 689 (5%). The Refugee Camp has an Early Child Development (ECD) Centre, Tongogara Primary School, St. Michael’s Secondary School and a vocational training center. Tongogara Refugee Camp Clinic is the only health facility within the camp and provides all basic primary care services free of charge.

Tongogara refugee camp falls under the agro-ecological zone five that normally experiences low amount of rainfall, on average 200–400 mm per year. The camp which occupies an area of about 1420 ha is bordered by Save River and Save Wildlife Conservancy. While electricity is available at TRC clinic, for lighting and cooking, refugees are largely provided with solar lamps and firewood. Site planning in the camp has been undermined by spontaneous and at times unlawful construction of houses by residents resulting in overcrowding particularly in areas that had access to electricity, mainly through illegal connections. The camp is characterized by gravel roads and access is difficult during the rainy season. Refugees at Tongogara survive on food assistance from the World Food Programme (WFP) and engage in horticulture, fishing, and livestock farming. Several tuck-shops have also been established within the camp. The UNHCR is the leading non-governmental organization (NGO) providing humanitarian assistance and partners with other organizations including Terre des Hommes (TDH), World Vision, National Organization for the Development of the Disadvantaged (NODED) and the Jesuit Refugee Service.

### Study population

The population included any individual residing within Tongogara Refugee Camp that was willing to participate in the study. Children under 12 years of age were interviewed with the assistance of their care givers. Any individual who was not resident in TRC during the outbreak period was excluded. Individuals who had symptoms suggestive of malaria but had no diagnostic test or laboratory confirmation were also excluded from the study. A minimum sample size of 78 cases and 78 controls was calculated using Fleiss formula embedded in Stat Calc function of Epi Info at 95% confidence interval and 80% power. The calculation was based on a study by Masango et al. in Zimbabwe, where those living in a house with open eaves had 8.7 higher odds of contracting malaria and 36/63 (57%) of controls were living in a house with open eaves [13].

### Sampling technique

All confirmed cases from the 22nd of March 2021 to the 18th of April 2021 were included in the study. A line-list was created using the T12 and Integrated management of childhood illnesses (IMCI) registers at TRC clinic. The sampling frame for controls was the T12 and IMCI registers. The line-list for controls was created for patients who sought health services at the camp clinic between the 22nd of March and 18th of April 2021 and had a negative malaria rapid diagnostic test. The total number of controls was 273. Each patient on the line list was allocated a number and simple random sampling was used to enroll the first control into the study using the Microsoft Excel software “RANDBETWEEN”. Systematic random sampling was used to select subsequent controls at a constant sampling interval of (N/n) = 3, where the total number of controls was N = 273 and the sample size required was n = 80.

### Data collection methods

Interviewer-administered questionnaires were used to collect information on demographic and behavioural characteristics, risk factors for contracting malaria, knowledge of malaria and practices among cases and controls at household level. Information on malaria education received in the last three months, defined as any health talks related to malaria received three months prior the clinic visit was collected. Three Community Health Promoters working as Community-Based Health Workers within the camp assisted with language translation during the interviews between English and other languages common among respondents (i.e. Swahili, French, Shona and Portuguese). A key informant guide was also used to elicit information on malaria outbreak preparedness and response. Key informants interviewed at TRC clinic included the Sister-in-charge, three Nurses, one Environmental Health Technician (EHT), one Pharmacy Technician, and four Community Health Promoters. The District Environmental Health Officer (DEHO) for Chipinge District was also interviewed.

Health records were reviewed to assess how cases were managed by going through outpatient cards and clinic records. The surrounding environment was assessed by observation for potential larval habitats close to the homes. The anopheles mosquito larvae characteristically rest parallel to and just below the water surface. Observations were done as interviews were being conducted, with consent from the participants to check on whether the LLINs were available and hanged appropriately. A camera was used to take pictures of known environmental elements that facilitate breeding of anopheles mosquitoes in the camp site such as stagnant water bodies, water filled pits, and swamps. The availability of resources, outbreak preparedness and response was assessed using a customized checklist. The checklist was applied to the sister in charge at TRC clinic to assess the adequacy of medicines and diagnostic test kits at the clinic. The presence of information, education and communication (IEC) material in the camp and the adequacy of health workers at the TRC clinic were also assessed.

### Field testing the questionnaire

To check for the validity and reliability of the data collection tool the questionnaire for cases and controls was pre-tested at TRC clinic on four patients that were conveniently enrolled whilst seeking postnatal care services which was five percent of the calculated sample size. The respondents were informed that the exercise was a field test being conducted to improve the data collection instrument for a study. Questions that were difficult for participants to comprehend as well as missing responses to close-ended questions were edited before the final duplication of the questionnaire.

### Data analysis

Epi Data version 3.1 was used for data entry and Epi Info™ version 7.2.4 was used for quantitative analysis to generate medians, frequencies, proportions, odds ratios and their corresponding 95% confidence intervals. Forward step-wise logistic regression analysis was done to determine the independent factors associated with contracting malaria. All variables that were associated with contracting malaria with a p-value ≤ 0.25 were included in the logistic regression model. For all the tests conducted, p < 0.05 indicates statistical significance. Data from key informants was analyzed thematically following steps by Creswell and Creswell, for coding qualitative data manually to identify themes [14]. Clusters of comments from responses to questions were identified and arranged into topics. Each topic was assigned a descriptive code and sorted into themes. Knowledge levels on malaria were assessed among study participants using a 3 point Likert scale where those who scored zero to two questions correctly had poor knowledge, those who scored three had fair knowledge and those who scored four to five had good knowledge.

## Results

The malaria outbreak investigation included 80 cases and 80 controls. Most study participants were males 47 (59%) of cases and 43 (54%) of controls (*p* = 0.524). The median age for cases was 15 years [Interquartile range (IQR) = 9–26)], and controls, 17 years [Interquartile range (IQR) = 10–30)] was comparable (p = 0.755). The majority of cases were from Mozambique 36 (45%) followed by the Democratic Republic of Congo 29 (36%). No participants had been resident in the camp for < 6 months with 57 (71%) cases and 56 (70%) controls having been resident in the camp for a period of < 5 years (p = 0.233). About 45 (56%) cases and 30 (37%) controls had primary education as the highest level of education attained (p = 0.017). Most cases 61 (76%) as well as controls 52 (65%) were not involved in any income generating activities (p = 0.295). Table 1 summarizes the socio demographic characteristics of study participants at TRC.

**Table 1:** Socio-demographic characteristics of study participants at Tongogara Refugee Camp, Chipinge District, Zimbabwe, 2021

Variable	Category	Cases		Controls		p-value
		n = 80	%	n = 80	%	
Sex	Male	47	59	43	54	0.463
	Female	33	41	37	46	
Age (years)	≤ 5 years	12	15	10	12	0.693
	> 5 years	67	84	67	84	
	Age not known	1	1	3	4	
		Median age = 15 Q1 = 9; Q3 = 26		Median age = 17 Q1 = 10; Q3 = 30		0.755
Country of origin	Mozambique	36	45	36	45	0.97
	DRC	29	36	27	34	
	Zimbabwe	6	8	5	6	
	Burundi	4	5	5	6	
	Rwanda	3	4	3	4	
	Other	2	2	4	5	
Level of education	None	19	24	23	29	0.017
	Primary	45	56	30	37	
	Secondary	9	11	23	29	
	Tertiary	7	9	4	5	
Occupation	None	61	76	52	65	0.295
	Farming	9	11	13	16	
	Shop attendant	4	5	2	3	
	Other	6	8	13	16	
Period of residence in the camp (years)	2–5	57	71	56	70	0.233
	6–10	10	13	11	13	
	11–15	8	10	9	11	
	16–20	5	6	5	6	

Most cases 70 (87%) and controls 65 (81%), resided within 3 km of a stagnant water body (p = 0.276). Majority of cases 67 (84%) and 68 (85%) controls did not have a mosquito net and mentioned that nets which had been distributed to them more than three years prior to the study were torn (p = 0.043). About 42 (53%) controls resided in refugee housing units while 41 (51%) of cases lived in houses constructed with bricks and iron roof sheets (p < 0.001). Indoor residual spraying using pirimiphos methyl had been conducted in the last 12 months for 68 (85%) cases and 70 (88%) controls (p = 0.646). New arrivals stay in the transit section of the camp. After the administrative processes which include registration and issuing of personal identification documents, refugees receive the core relief package which includes shelter and domestic items. Forty families that arrived after the IRS period did not receive mosquito nets and their houses were scheduled for the next IRS exercise.

Most cases 48 (60%) engaged in outdoor activities at night compared to 23 (29%) controls (p < 0.001). Most commonly mentioned outdoor activities included socializing with friends and family, playing, cooking and sleeping outside during the hot summer nights. Based on self-reported travel 71 (89%) cases and 77 (96%) controls self-reported had not travelled outside the camp in the last month (p = 0.086) Key informant interviews suggested that there was illegal cross-border travel among Mozambican Nationals who would return to their homes during peak agricultural seasons to plant and harvest crops, however, this could not be verified (Table 2).

**Table 2:** Household and behavioural characteristics of study participants at Tongogara Refugee Camp, Chipinge District, Zimbabwe, 2021

Variable	Category	Cases		Controls		p-value
		n = 80	%	n = 80	%	
Presence of a stagnant water body 3 km from the home	Yes	70	87	65	81	0.276
	No	10	13	15	19	
Slept under a mosquito net the previous night	Yes	10	13	11	14	0.815
	No	70	87	69	86	
Ownership of a mosquito net in the last 6 months	Yes	13	16	12	15	0.087
	No	67	84	68	85	
Type of house	Brick under iron	41	51	34	42	< 0.001
	Brick under asbestos	18	22	4	5	
	Refugee housing unit	14	18	42	53	
	Mud under thatch	7	9	0	0	
Holes or open eaves in the house	Yes	26	33	10	13	0.002
	No	54	67	70	87	
IRS in the last 12 months	Yes	68	85	70	87	0.646
	No	12	15	10	13	
Outdoor activities at night	Yes	48	60	23	29	< 0.001
	No	32	40	57	71	
Wearing clothes that cover during outdoor activities	Yes	26	32	56	70	< 0.001
	No	54	68	24	30	
Use of mosquito repellents	Yes	10	13	3	4	0.043
	No	70	87	77	96	
Travel outside the camp (self-reported)	Yes	3	4	9	11	0.086
	No	77	96	71	89	
Malaria education in the last 3 months	Yes	9	11	13	16	0.463
	No	71	89	67	84	

### Malaria cases by time

**Fig. 1:**
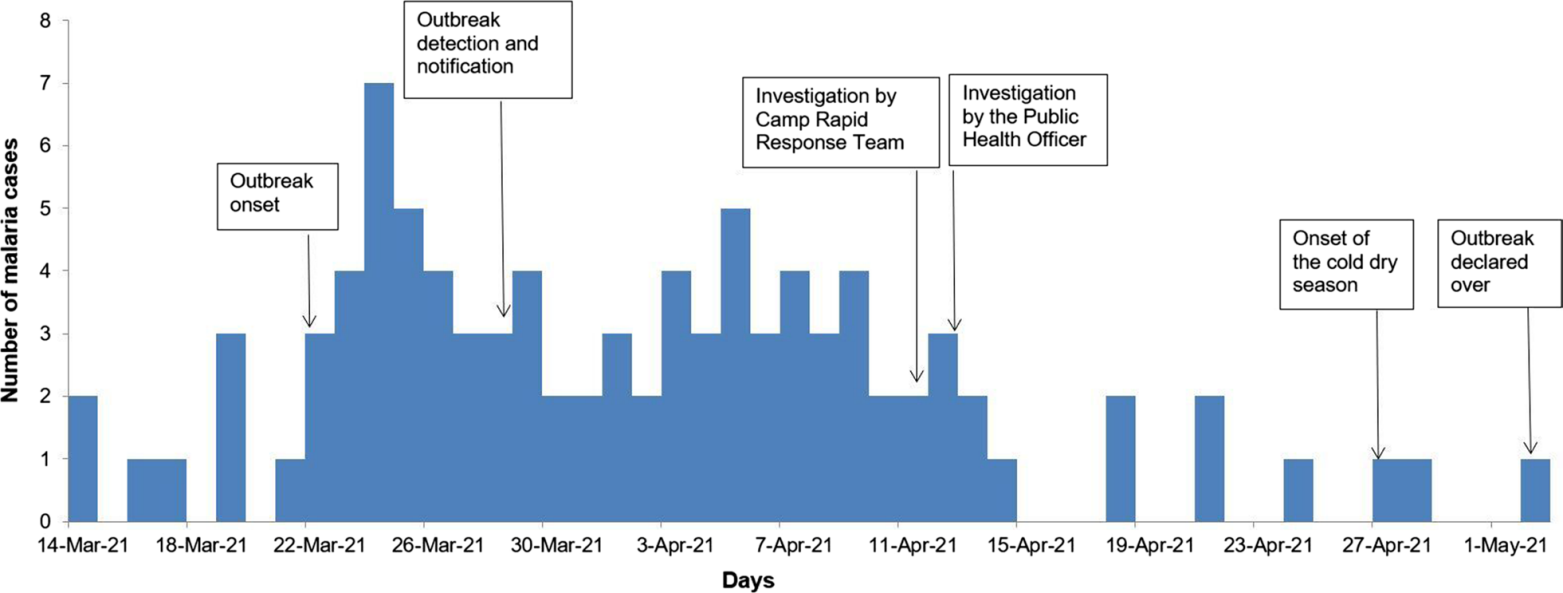
Epicurve for malaria cases at Tongogara refugee camp Zimbabwe, 2021

Tongogara refugee camp clinic recorded an increase in malaria cases above the alert and action threshold limits in week 12 of 2021 reaching a peak of 29 cases by the end of week 12. Thereafter, the cases declined steadily. The decline in the number of malaria cases in week 17 also coincided with the start of the winter season by the end of the month of April. The epicurve for malaria cases at TRC, Chipinge District, Zimbabwe 2021 is based on the date of diagnosis (Fig. 1).

### Malaria cases by place

**Fig. 2:**
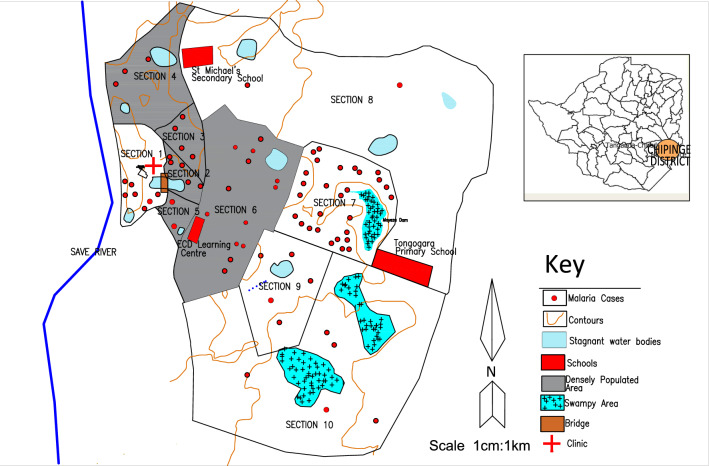
Spot map showing distribution of malaria cases in Tongogara refugee camp Zimbabwe, 2021

Malaria cases were distributed throughout the 10 residential sections within TRC, however, most cases 28 (35%) came from section 7 followed by section 6 which had 13 (16%) and section 9 with 11 (14%). Mayeza Dam, which is by definition a marsh characterised by wetland frequently or continually inundated with water and typically has grasses and herbaceous plants was observed in section 7. A swampy area was also observed in section 10. Stagnant water bodies were a common feature around the camp. A bridge was constructed between section 1 and 2 near TRC clinic and stagnant water could be seen on both sides of the bridge. Figure 2 illustrates the spot map showing the distribution of malaria cases during the outbreak.

### Environmental assessment

**Fig. 3:**
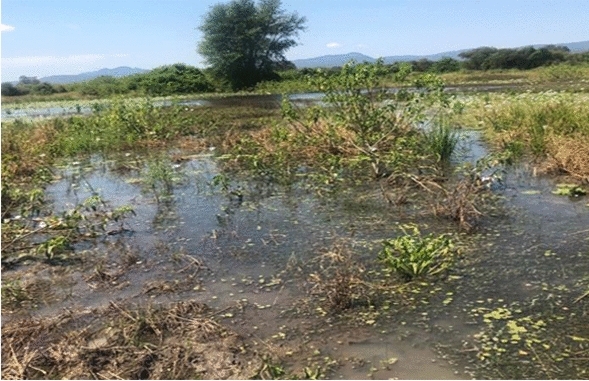
Mayeza Dam in section 7 Tongogara refugee camp, Zimbabwe, 2021

**Fig. 4:**
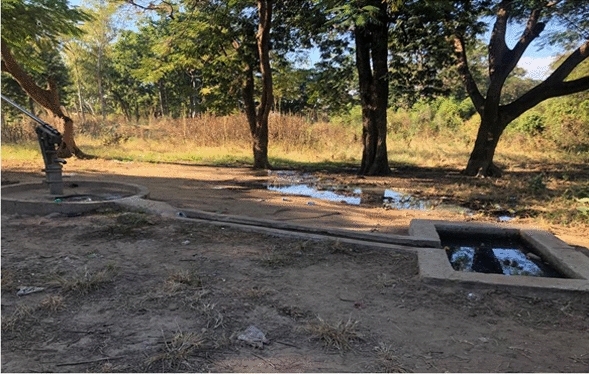
Stagnant water from communal tap collecting in a ditch at Tongogara refugee camp Zimbabwe, 2021

**Fig. 5:**
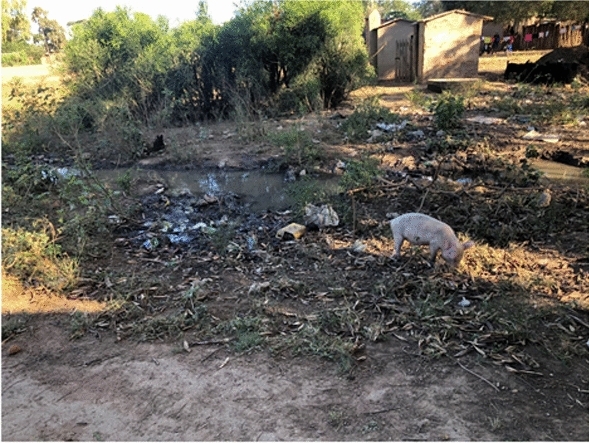
Stagnant water from receding floods, litter, and bushes near homesteads at Tongogara refugee camp Zimbabwe, 2021

Natural vector breeding sites observed included swampy areas in section 10 and Mayeza Dam in section 7 (Fig. 3). Vector breeding sites from human activities included water from the communal taps collecting in reservoirs or ditches (Fig. 4). An irrigation scheme within the camp as well as pits created by residents as water reservoirs for gardening and waste from the piggery project also provided potential vector breeding sites. Poor waste management practices were observed as there were plastic containers and liter around the camp and in the stagnant water bodies (Fig. 5). Key informants highlighted that there was poor drainage in the camp.

### Malaria case management

All cases were diagnosed at TRC clinic using the First Response Malaria Antigen (pLDH/HRP_2_) Combo rapid diagnostic test (RDT) following Ministry of Health standardized protocols. Cases presenting with mild symptoms were 43 (54%) while 33 (41%) had moderate symptoms and 4 (5%) presented with severe symptoms. Most cases 64 (80%) presented with a fever followed by a headache and chills constituting 52 (65%) and 30 (38%), respectively. There were no complicated malaria cases, and all patients were treated appropriately with co-artemether at TRC clinic according to the national malaria treatment guidelines for weight and age. No malaria deaths were reported in the camp. Most cases 78 (97%) had completed their course while 2 (3%) stopped taking their medication when the symptoms subsided. Apart from malaria treatment guidelines, there was no IEC material on malaria displayed at the clinic.

### Outbreak emergency preparedness and response at Tongogara Refugee Camp Clinic

Tongogara refugee camp clinic has four nurses, one environmental health technician, one pharmacy technician and one nurse aide. An assessment of the outbreak emergency preparedness and response showed that five out of seven health workers had received previous training on malaria case management. Two nurses received training on Integrated Disease Surveillance and Response (IDSR). The remaining five health workers were not trained due to limited resources.

The emergency preparedness and response committee comprising of the camp administrator, camp coordinator, nurse, environmental health technician and a World Vision representative meets once every month. An emergency preparedness and response plan was available at TRC clinic. The malaria case management guidelines and weekly malaria threshold limits were up to date and displayed on the walls, however, there was no spot map or line list at the clinic. The malaria outbreak was detected 7 days after the onset of the outbreak and the clinic notified the outbreak to the district within the same period. However, the Rapid Response Team (RRT) initiated an outbreak investigation after 3 weeks due to limited Environmental health technicians. The district RRT investigated the outbreak 2 months after the outbreak had occurred. Long-lasting insecticidal nets (LLINs) are routinely distributed to women once during their pregnancy. The IRS coverage for TRC was 85% compared to the program reported coverage of 94%. No larviciding was conducted at the refugee camp. Table 3 shows the malaria outbreak emergency preparedness and response at TRC.

**Table 3:** Malaria outbreak emergency preparedness and response at Tongogara Refugee Camp Chipinge District, Zimbabwe, 2021

Activity	Target	Achieved
Number of health workers trained		
Integrated disease surveillance and response	5	2
Malaria case management	7	5
Emergency preparedness		
Availability of standard treatment protocols for malaria	Available	Available
Rapid Response Team (RRT)	1 team available	1 team available
EPR plan	Available	Available
Outbreak detection and notification		
Outbreak detection, notification and response	Within 48 h	Untimely: Outbreak was detected after 7 days and notified after 14 days
Initiation of investigation	Within 48 h	Untimely, the RRT initiated the investigation 3 weeks after the outbreak was detected
Line list	Available	Not available
Spot map	Available	Not available
Outbreak control		
Outbreak control	14 days	The outbreak persisted for 28 days
Community sensitization and health education	Done	Done
Distribution of mosquito nets in the last 6 months	85%	2%
IRS (population coverage)	95%	85%
Insecticide efficacy testing in the last 6 months	Done	Not done
Larviciding	95%	0%

### Entomological surveillance activities conducted in Tongogara Refugee Camp

Entomological surveillance activities conducted at the refugee camp involved larval sampling and pyrethrum spray catches to determine the indoor vector density and distribution. Pyrethrum spray catches (PSCs) conducted in November 2020 in section 7 and section 10 of the camp before the IRS exercise (pre-IRS PSCs) captured eight *Anopheles gambiae* and 66 culicines. However, post-IRS PSCs did not capture any vectors. No further evaluations for vector density comparison were done due to limited supply of aerosol to use. To further investigate the outbreak PSCs were performed in section 7 in June 2021 and did not yield any indoor resting mosquitoes, however larval sampling conducted in response to the outbreak at Mayeza Dam in section 7 harvested 18 Anopheles larvae**.**

### Factors associated with contracting malaria at Tongogara Refugee Camp, Chipinge District, Zimbabwe, 2021

**Fig. 6:**
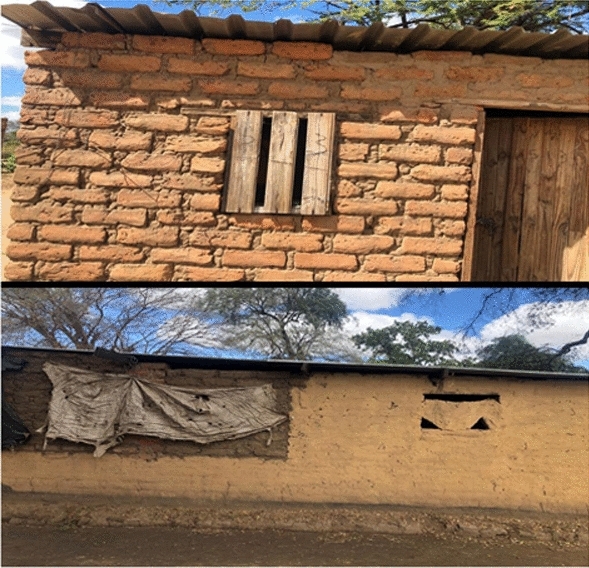
Brick houses under iron sheets with open eaves, holes and open makeshift windows at Tongogara refugee camp Zimbabwe, 2021

The odds of contracting malaria were 3.37 times higher when residing in a house with holes or open eaves compared those who did not**.** While some household had open eaves, others had makeshift windows that could not be closed (Fig. 6).

The odds of contracting malaria were 3.72 times higher for those engaging in outdoor activities at night when compared to those who did not [OR = 3.72 (95% CI, 1.92–7.19)]. The odds of contracting malaria were 4.76 times higher for those who did not wear clothes that covered their whole body while engaging in outdoor activities at night when compared to those who wore clothes that covered the whole body [OR = 4.76 (95% CI, 2.43–9.30)]. The odds of contracting malaria when residing in a refugee housing unit were 72% lower compared to participants residing in a house with brick under iron and was considered protective [OR = 0.28 (95% CI, 1.15–12.08)]. Table 4 shows the factors associated with contracting malaria at TRC, Chipinge District, Zimbabwe, 2021.

**Table 4:** Factors associated with contracting malaria at Tongogara Refugee Camp, Chipinge District, Zimbabwe, 2021

Variable	Cases		Controls		OR	(95% CI)	p-value
	n = 80	%	n = 80	%			
No. of children ≥ 5 years in household							
0	67	84	53	67	Reference		
1	7	9	10	7	0.55	0.20–1.55	0.261
2 +	6	7	17	6	0.28	0.10–0.76	0.012
Type of house							
Brick under iron	41	51	34	42	Reference		
Pole/mud under thatch	7	9	0	0	–	–	–
Refugee housing unit	14	18	42	53	0.28	(0.13–0.59)	0.001
Brick under asbestos	18	22	4	5	3.73	(1.15–12.08)	0.028
Holes or open eaves in the house							
No	54	68	70	87	Reference		
Yes	26	31	10	13	3.37	(1.5–7.58)	0.003
Outdoor activities at night							
No	32	40	57	71	Reference		
Yes	48	60	23	29	3.72	(1.92–7.19)	< 0.001
Wear clothes that cover the body during outdoor activities at night							
Yes	26	32	56	70	Reference		
No	54	68	24	30	4.76	(2.43–9.3)	< 0.001

### Independent risk factors associated with contracting malaria at TRC, 2021

The independent risk factors associated with contracting malaria among study participants at TRC were engaging in outdoor activities at night [AOR = 2.74 (95% CI 1.04–7.22) and wearing clothes that do not cover the whole body [AOR 4.26 (95% CI, 1.43–12.68)] during outdoor activities at night. Residing in a refugee housing unit (RHU) reduced the risk of contracting malaria by 82% [AOR = 0.18 (CI, 0.06–0.55)].

### Malaria knowledge among study participants at TRC, Zimbabwe, 2021

Amongst the study participants, 58 (71%) of cases knew how malaria was transmitted compared to 74 (91%) controls (p = 0.029). Majority of cases 50 (63%) knew that malaria was common during the rainy season compared to 71 (89%) controls (p < 0.01). Sleeping under a mosquito net was the most commonly mentioned protective measure suggested by 59 (74%) cases and 67 (84%) controls, however, only 18 (23%) cases 21/80 (26%) controls mentioned IRS as one of the malaria preventive measures. Overall, 33/80 (41%) cases had poor knowledge on malaria compared to 23/80 (29%) of controls (p = 0.097). There was a statistically significant difference between fair knowledge levels as 18/80 (19%) of cases had fair knowledge on malaria compared to 28/80 (35%) of controls (p = 0.02).

## Discussion

The independent risk factors associated with contracting malaria among study participants at TRC were engaging in outdoor activities at night and wearing clothes that did not cover the whole body during outdoor activities. Outdoor activities after sunset, such as cooking and socializing expose one to mosquito species that typically feed at dusk and dawn or at night. This is consistent with findings by Mugwagwa et al., who found that outdoor activities after dusk were significant risk factors for contracting malaria, while wearing clothes that cover the whole body was protective [15].

Residing in a refugee housing unit (RHU) was found to decrease the risk of contracting malaria. During the hot summer nights refugees reported that they would occasionally sleep outside their houses. The RHU is a durable temporary home and has windows that are covered by breathable material which offers protection from insects when windows are open. The RHU design provides the recommended minimum living space for a small family, however, it has an average lifespan of 3 years [16]. Therefore, it remains necessary to provide more sustainable long-term housing structures for refugees, the majority of whom had been in the camp for at least 2 years.at the time of the study.

Residing in a house with holes or open eaves increased the risk of contracting malaria. Gaps provide an entry route for mosquitoes into to the house which increases the risk of contracting malaria. These findings are consistent with Mundagowa and Chimberengwa, who found that sleeping in a poorly constructed house with open eaves increased malaria risk [17]. Yaro et al. concluded that improving the construction of houses to include screened windows, closing eaves and solid roofs may reduce malaria incidence [18].

The majority of cases resided in section 7 which is predominantly occupied by Mozambican nationals. Despite constituting approximately one-tenth of the total population, nationals from Mozambique accounted for almost half of malaria cases. Unverified reports suggested that Mozambican nationals would illegally return to their country to plant and harvest crops. Mozambique has a high malaria burden, accounting for 4% of malaria cases globally [1]. Mayeza Dam in section 7 from which Anopheles mosquito larvae were harvested suggests that the dam may have been a major environmental source of the mosquito vectors compared to all the other sections within the camp thus accounting for the higher number of cases.

Tongogara Refugee Camp is bordered by Save River on the West. Additionally, heavy rains received between January and March of 2021 resulted in the creation of stagnant water bodies in ditches and marshes increasing the number of potential malaria vector breeding sites which may have resulted in increased vector density. This is consistent with findings by Workineh et al., who found that the presence of rivers, stagnant water and poor environmental controls were associated with occurrence of malaria outbreaks [19]. The WHO recommends larviciding in areas where mosquito breeding sites are few, fixed and findable, as is the case at the camp [6].

Poor drainage in the camp exacerbates the development of stagnant water bodies. The Roll Back Malaria Vector Control Working Group report of 2017 found that soil with poor drainage enabled larval habitats for *Anopheles arabiensis* mosquitoes at Doro refugee camp in South Sudan [20]. Resources to facilitate the use of drone technology to carry out hydrological mapping to facilitate planning for improved drainage in the camp are limited.

Human activities involving agricultural activities, water from communal taps collecting in ditches and poor waste management practices also resulted in potential vector breeding sites.

This is consistent with findings by Bayoh et al., who observed larval habitats from man-made pits of water associated with tap stands installed for water delivery to residents in the camp [21].

Tongogara Refugee Camp achieved an optimal IRS coverage which meets the WHO recommended impact level of 85% but failed to reach the national target of 95%. However, the benefits of this intervention are minimized when refugees engage in outdoor activities at night. Compared to the rest of Chipinge District which uses the organochlorine insecticide (DDT), broad spectrum insecticides such as organophosphates (pirimiphos methly) or pyrethroids are used for IRS in Tongogara Refugee Camp. Organochlorine insecticide has a slow knockdown effect on mosquitoes but long efficacy of 9–12 months while organophosphates have a fast knockdown effect on mosquitoes and other insects including fleas, lice, cockroaches and termites. This increased acceptability of IRS among refugees consistent with Messenger et al., who found that other benefits of vector control interventions, such as reducing flies in households and nuisance biting may make IRS interventions more acceptable to the community [4]. To avoid insecticide resistance, the Zimbabwe 2016–2020 National Malaria Strategic Plan, recommends the rotation of insecticides after two years of use and the use of insecticides with different modes of action taking into consideration the available vector resistance data [11].

The majority of study participants did not own a mosquito net and refugees who had received a net 3 years prior to the study reported that the nets were torn. Long lasting insecticidal nets (LLINs) are designed to retain their efficacy against mosquito vectors for a minimum of 3 years or 20 standard washes under laboratory conditions [22, 23]. The WHO recommends the provision, use and timely replacement of LLINs [24]. While this is core intervention, LLINs are only effective if used consistently, hence the need for continued health education [17]. The provision of nets for outdoor spaces in combination with IRS among this population may be effective in reducing the incidence of malaria.

Despite the outbreak being detected after 48 h, TRC clinic was well prepared to respond to the outbreak with adequate stocks of diagnostic test kits and anti-malarial medicines. The RRT in the camp delayed the outbreak investigation due to limited human resources as there is one resident environmental health technician who is not replaced when off duty. Malaria cases are reported almost weekly at TRC clinic, which may have also delayed investigating the increase in malaria cases above threshold levels. The response to the outbreak by the district was not timely as entomological surveillance activities were conducted during the winter season in June when temperatures were low and some of the larval habitats had dried up interrupting the anopheles mosquito lifecycle. This is consistent with findings by Muchena et al. who concluded that poor outbreak response may propagate malaria outbreaks [25].

All patients received the appropriate treatment, according to national guidelines at the clinic, however, a few participants reported that they had stopped taking their medication when malaria symptoms had subsided which may show a gap in malaria health education. Contrary to these findings, Bawate et al. found sub-optimal adherence to national malaria treatment guidelines in Uganda [26].

The majority of cases had poor knowledge on malaria and the education level of the cases was significantly lower than the controls. A higher level of education results in improved knowledge and consequently impacts behavioural practices on malaria prevention. Our findings are consistent with Kureya et al. who found that having poor knowledge predisposed people to contracting malaria [27]. There is need to conduct a community needs assessment and deliver targeted health promotion messages and IEC material that address socio-demographic, sociocultural and linguistic variations among the refugees that can be delivered by CBHWs. Addressing knowledge gaps through campaigns particularly before the onset of the malaria season may facilitate successful adoption of malaria prevention measures in the community.

### Limitations

The limitations of the study were that it involved the use of local community health promoters as interpreters. Although the interview questions were first piloted in the non-English speaking participants’ languages mainly Swahili, French, Shona and Portuguese, interpreters were relied upon to keep the questions non-leading and neutral as well as to prompt the participants for further clarification in some instances. Using the community health promoters who were already known by the community compromised confidentiality as there was a third party listening to the interview. Recall bias may be a limitation in our study as participants were responding to questions about the past. Importation of malaria cases from Mozambique could not be ascertained in this study.

## Conclusion

The malaria outbreak at TRC reemphasizes the role of behavioural factors in malaria transmission. Independent risk factors associated with contracting malaria were engaging in outdoor activities at night and wearing clothes that do not cover the whole body during outdoor activities at night. The presence of stagnant water bodies, particularly Mayeza Dam, poor environmental controls and delayed outbreak response propagated the outbreak at Tongogara Refugee Camp. Community assessment on knowledge, attitudes and practices of refugees is recommended to facilitate intensified and targeted health education to address human behaviours that expose one to malaria. We recommend the distribution of mosquito repellents and outdoor mosquito nets for use during outdoor activities at night. We also recommend larviciding of existing vector breeding sites.

### Public health action

Health education was given to study participants at the end of each interview so as to demystify myths and correct misconceptions on malaria transmission, prevention and control. Participants who had stopped taking their anti-malarial medicine were referred to the clinic.

## Data Availability

Data for this study have been included within the document. For any further information that might be required, the corresponding author is willing to provide the information.

## Abbreviations

ACT: Artemisinin-based combination therapy
DEHO: District Environmental Health Officer
EHT: Environmental health technician
IRS: Indoor residual spraying
IDSR: Integrated disease surveillance and response
IQR: Interquartile range
LLINs: Long-lasting insecticidal nets
PSCs: Pyrethrum spray catches
RDNS: Rapid disease notification system
TRC: Tongogara refugee camp
UNHCR: United Nations High Commissioner for Refugees

## References

[1] WHO. World malaria report 2020 [Internet]. Geneva, World Health Organization, 2020. Available from: https://www.who.int/publications/i/item/9789240015791.

[2] WHO. Fact sheet about malaria [Internet]. Geneva, World Health Organization, 2021 [cited 2021 Apr 15]. Available from: https://www.who.int/news-room/fact-sheets/detail/malaria.

[3] President’s Malaria Initiative, Zimbabwe. CDC Global Health [Internet]. 2020 [cited 2021 Apr 16]. Available from: https://www.cdc.gov/globalhealth/countries/zimbabwe/annual-report/pmi.html.

[4] Messenger LA, Furnival-Adams J, Pelloquin B, Rowland M (2020). Vector control for malaria prevention during humanitarian emergencies: protocol for a systematic review and meta-analysis. medRxiv..

[5] WHO. Malaria control in complex emergencies: an inter-agency field handbook. Geneva, World Health Organization, [Internet]. Available from: https://apps.who.int/iris/handle/10665/43383.

[6] Anderson J, Doocy S, Haskew C, Spiegel P, Moss WJ (2011). The burden of malaria in post-emergency refugee sites: a retrospective study. Confl Health.

[7] Office for Coordination of Humanitarian Affairs. Malaria Fact Sheet. No. 94. [Internet]. ReliefWeb, 2014. Available from: https://reliefweb.int/report/world/malaria-fact-sheet-no-94-updated-march-2014.

[8] de Mendonça VR, Barral-Netto M (2015). Immunoregulation in human malaria: the challenge of understanding asymptomatic infection. Mem Inst Oswaldo Cruz.

[9] WHO (2013). Malaria control in humanitarian emergencies: an inter-agency field handbook.

[10] Centers for Disease Control and Prevention (CDC). Where malaria occurs [Internet]. 2020. [cited 2021 Apr 16]. Available from: https://www.cdc.gov/malaria/about/distribution.html.

[11] U.S. President’s Malaria Initiative. Zimbabwe Malaria Operational Plan FY [Internet]. 2020 [cited 2021 Jun 16]. Available from: https://www.pmi.gov/docs/default-source/default-document-library/malaria-operational-plans/fy20/fy-2020-zimbabwe-malaria-operational-plan.pdf?sfvrsn=6.

[12] Meteorological Services Department of Zimbabwe National Climate Outlook Forum [Internet]. [cited 2021 Jun 16]. Available from: http://www.cfuzim.com/wp-content/uploads/2020/09/msd2021.pdf.

[13] Masango TT, Nyadzayo TK, Gombe NT, Juru TP, Shambira G, Chiwanda S (2020). Factors associated with malaria infection in Mudzi District, Mashonaland East Zimbabwe, 2019: a case-control study. BMC Public Health.

[14] Creswell JW, Creswell DJ. Qualitative methods. In: Research design: Qualitative, quantitative, and mixed methods approaches [Internet]. Thousand Oaks, CA: SAGE Publications; 2018. p. 183–213. Available from: https://researchskillsgmm.files.wordpress.com/2014/10/research-design-creswell-chapter-9.pdf.

[15] Mugwagwa N, Mberikunashe J, Gombe NT, Tshimanga M, Bangure D, Mungati M (2015). Factors associated with malaria infection in Honde valley, Mutasa district, Zimbabwe, 2014: a case control study. BMC Res Notes.

[16] Refugees UNHC for. Refugee Housing Unit - Fact Sheet [Internet]. UNHCR. [cited 2021 Jun 17]. Available from: https://www.unhcr.org/en-lk/getinvolved/fundraising/5c1127d24/refugee-housing-unit-fact-sheet.html.

[17] Mundagowa PT, Chimberengwa PT (2020). Malaria outbreak investigation in a rural area south of Zimbabwe: a case–control study. Malar J.

[18] Yaro JB, Tiono AB, Sanou A, Toe HK, Bradley J, Ouedraogo A (2021). Risk factors associated with house entry of malaria vectors in an area of Burkina Faso with high, persistent malaria transmission and high insecticide resistance. Malar J.

[19] Workineh B, Mekonnen FA, Sisay M, Gonete KA (2019). Malaria outbreak investigation and contracting factors in Simada District, Northwest Ethiopia: a case–control study. BMC Res Notes.

[20] Vector-Control-Humanitarian-Emergency-meeting-report-.pdf [Internet]. [cited 2021 Apr 17]. Available from: https://endmalaria.org/sites/default/files/Vector-Control-Humanitarian-Emergency-meeting-report-.pdf.

[21] Nabie Bayoh M, Akhwale W, Ombok M, Sang D, Engoki SC, Koros D (2011). Malaria in Kakuma refugee camp, Turkana, Kenya: facilitation of *Anopheles arabiensis* vector populations by installed water distribution and catchment systems. Malar J.

[22] de Sousa JO, de Albuquerque BC, Coura JR, Suárez-Mutis MC (2019). Use and retention of long-lasting insecticidal nets (LLINs) in a malaria risk area in the Brazilian Amazon: a 5-year follow-up intervention. Malar J.

[23] Brooks HM, Paul MKJ, Claude KM, Mocanu V, Hawkes MT (2017). Use and disuse of malaria bed nets in an internally displaced persons camp in the Democratic Republic of the Congo: A mixed-methods study. PLoS ONE.

[24] WHO (2015). Global Malaria Programme. Global technical strategy for malaria, 2016–2030.

[25] Muchena G, Gombe N, Takundwa L, Tshimanga M, Bangure D, Masuka N (2017). Factors associated with contracting malaria in Ward 29 of Shamva District, Zimbabwe, 2014. South Afr Med J.

[26] Bawate C, Callender-Carter ST, Nsajju B, Bwayo D (2016). Factors affecting adherence to national malaria treatment guidelines in management of malaria among public healthcare workers in Kamuli District, Uganda. Malar J.

[27] Kureya T, Ndaimani A, Mhlanga M (2017). Malaria outbreak investigation in Chipinge, Zimbabwe: a case-control study. Iran J Parasitol.

